# Thin-Layer Chromatography (TLC) in the Screening of Botanicals–Its Versatile Potential and Selected Applications

**DOI:** 10.3390/molecules27196607

**Published:** 2022-10-05

**Authors:** Teresa Kowalska, Mieczysław Sajewicz

**Affiliations:** Institute of Chemistry, University of Silesia, 9 Szkolna Street, 40-006 Katowice, Poland

**Keywords:** TLC screening of plants, TLC screening of psychoactive plants, TLC-direct bioautography, effect-directed detection, chemotaxonomy of plants, quality control of medicinal plants, quality control of alimentary products, quality control of cosmetic products

## Abstract

The aim of this paper is to present a comprehensive overview of the main aims and scopes in screening of botanicals, a task of which thin-layer chromatography (TLC) is, on an everyday basis, confronted with and engaged in. Stunning omnipresence of this modest analytical technique (both in its standard format (TLC) and the high-performance one (HPTLC), either hyphenated or not) for many analysts might at a first glance appear chaotic and random, with an auxiliary rather than leading role in research, and not capable of issuing meaningful final statements. Based on these reflections, our purpose is not to present a general review paper on TLC in screening of botanicals, but a blueprint rather (illustrated with a selection of practical examples), which highlights a sovereign and important role of TLC in accomplishing the following analytical tasks: (i) solving puzzles related to chemotaxonomy of plants, (ii) screening a wide spectrum of biological properties of plants, (iii) providing quality control of herbal medicines and alimentary and cosmetic products of biological origin, and (iv) tracing psychoactive plants under forensic surveillance.

## 1. Introduction

Thin-layer chromatography both in its standard (TLC) and high-performance (HPTLC) format is known as a versatile and high-throughput liquid chromatography technique, with a wide range of important applications. These applications can roughly be divided into those in direct service of life sciences (such as botany, phytochemistry and medicine, and handling rather fundamental issues such as contributing to chemotaxonomy of plants, or searching for enzyme inhibitor templates) and the more practical goals. Practical applications are usually made on the demand of different regulatory bodies closely related to authentication and quality control of herbal medicines, alimentary herbs and spices, and they include forensic control of these ethnobotanicals, which as illicit highs, are subject to criminal law.

Among the most often employed screening TLC procedures, we find those which focus on drug control of synthetic pharmaceuticals, with special emphasis laid on substandard and fake drugs which are illegally traded in developing countries [1,2,3,4,5], yet this purely pharmaceutical issue is out of scope of our present overview. Construction of a chemistry-based taxonomy of plants is a meaningful help in plant systematics. Contribution of TLC is significant in this sense such that the thin-layer chromatographic screening of plant extracts facilitates identification of chemotaxonomic markers and the entire chemotaxonomic profiles, and hence, it facilitates determination of botanical taxa [6,7,8,9]. The TLC-based screening methods often target medicinal plants in the search for various different physiological properties of botanical material (e.g., the free radical scavenging, antimicrobial, and enzyme-inhibiting activity [10,11,12]). Hyphenation of TLC with other analytical techniques which allow for an in situ (i.e., directly on the chromatographic plate surface) recognition of biological properties of an individual compound or compound fraction has evolved into the so-called TLC-EDA strategy (with EDA held for the effect-directed analysis) [13,14], which in particularly favourable cases, might suggest novel structural motifs for synthetic medicines. Two more areas of application of the TLC-based screening approach are quality control of plant medicines, botanical alimentary and cosmetic products (e.g., [15,16,17]) and psychoactive ethnobotanicals, which in many cases are illicit and subject to criminal law. In the latter case however, the TLC methods of screening psychoactive botanicals sometimes tend to remain unpublished, or purposely provide incomplete analytical details (e.g., [18]).

It is not an aim of this paper to provide a traditional and exhaustive review on applications of TLC to screening botanicals in their various different roles and functions but instead, to provide a comprehensive blueprint equipped with a selection of experimental examples, which clearly define distinct competences of this versatile, efficient and high-throughput analytical technique. It is an intentional and perhaps a slightly provocative mosaic of the diverse TLC possibilities dispensed in a single paper and intended to make our readers feel a bit dizzy and, in that way, to capture their attention. It will also be an added value of this paper and an undoubted joy for its authors if it manages to light a spark of inspiration with some of those who are interested in plant analysis and who search for simple, reliable and cost-effective approaches in this field.

Last not least, we would like to share with our readers a reflection on the distinction made by many authors who divide their thin-layer chromatographic methods into two categories, i.e., belonging to the TLC in its standard version, or to the high-performance TLC (HPTLC). Citation of all working examples presented in the forthcoming sections of this review preserves original classification of the techniques (either TLC, or HPTLC), as declared in respective publications by their authors. However, no normative guidance is available thus far which might allow for an official distinction between TLC and HPTLC, and the most compelling argument is that the IUPAC Gold Book of chemical terminology does not mention HPTLC, but TLC only (see Orange Book, 2nd Edition). Basically, thin-layer chromatography is a separation technique, and the two major factors which rule separation are chemical and physical structure of stationary phases, so that the lower is the average diameter of the stationary phase particles of a given type, and the more regular their shape, the more efficient separation can be expected. To this effect, manufacturers of the TLC plates which are intended for analytical (and not for preparative) tasks denote them either as the TLC- or the HPTLC-type plates, and this differentiation should unequivocally define the technique itself. However, numerous practitioners of thin-layer chromatography firmly believe that using an advanced auxiliary instrumentation (e.g., an automatic sample applicator, densitometric scanner of the developed chromatograms, or video acquisition of the separation results) enhances the separation result obtained on the TLC-type chromatographic plates such that their method can justifiably be regarded as the HPTLC method. In our view, however, such logic is a kind of soft manipulation, such that in all modern planar chromatography laboratories, an advanced instrumentation is an obligatory precondition, so that at the very end, the stationary phase type alone (either the TLC- or the HPTLC-type phase) is the only decisive factor which considerably affects separation quality and unequivocally defines the technique.

## 2. Thin-Layer Chromatography in Chemotaxonomy of Plants

Although slightly more than one third of a million species of plants are known to humans today, taxonomy was recognized as a formal subject in the early 19th century only, and then, it was understood as the science of identifying, naming, and classifying plants. The earliest systems of plant classification, certainly used even in prehistorical times, were based on one or a few easily observable characters of plants, such as their habit (trees, shrubs, herbs, etc.) or floral characteristics (particularly the number of stamens and carpels). These classification systems were based on arbitrarily selected and easily observable features, and they are therefore viewed as artificial. Natural classification systems are based upon overall resemblances, mostly in gross morphology, thus, utilizing as many taxonomic characters as possible to group taxa. The most advanced classification systems also use as many taxonomic characters as possible yet in addition to phylogenetic (i.e., evolutionary) interpretations. Chemotaxonomy is an advanced approach to plant classification, which is based on plant biochemistry and chemistry, as it supplements morphological evidence at another (mostly molecular) level of structural organization. Within the framework of this approach, relations are investigated between the classes of plants and the occurrence of specific substances or substance groups in plant tissues [19,20]. Thin-layer chromatography has proved an excellent tool for fast screening of plant material for chemotaxonomic purposes by providing easy and reliable access to the plant fingerprints, which is a very important step in the chemotaxonomic procedure exerted with the aid of TLC. Relatively simple, inexpensive and fast TLC methods of fractionating complex plant extracts and obtaining respective TLC fingerprints permit for easy perceiving similarities among different plant species and make a solid base for the consecutive chemotaxonomic steps [21]. In the following paragraphs, selected examples are given of TLC contributions to the chemotaxonomy of plants.

The authors of [8] provided an example of the TLC application to solving a taxonomic problem with certain plants from the sandstone region of southern KwaZulu-Natal and Pondoland areas, identified as a distinct centre of endemism in South Africa and therefore called the Pondoland Centre (PC). This region abounds in species from the *Maytenus* genus (family of Celastraceae), out of which at least four species seem to be endemic to the region. Thus, an idea arose about a possible split of representatives of the endemic *Maytenus* genus into more natural and closely related complexes of species.

Knowing that a vast number of the secondary plant metabolites (such as sesquiterpenoids, triterpenoids, alkaloids and flavonoids) have earlier been isolated from the *Maytenus* genus specimens from around the globe, the authors decided to explore chemotaxonomic potential of the secondary metabolites fractions extracted from the leaves of the *Maytenus* genus representatives characteristic of the PC region and to fingerprint them by means of TLC. Thus, the leaves of fourteen *Maytenus* genus species originating from the PC region underwent an extraction with methanol, and then, the fingerprint analysis of the obtained extracts was carried out on the silica gel pre-coated chromatographic plates using four solvent systems of an increasing polarity (light petroleum–ethyl acetate (8:3, *v*/*v*), light petroleum–ethyl acetate chloroform–formic acid (8:7:5:1, *v*/*v*/*v*/*v*), chloroform–ethyl acetate–formic acid (5:4:1, *v*/*v*/*v*), and chloroform–methanol–water (12:3:1, *v*/*v*/*v*)), to obtain fingerprints targeting different groups of secondary metabolites. The chromatograms were acquired photographically. Based on these fingerprints, the authors succeeded in splitting certain *Maytenus* genus species into smaller and closely related complexes (e.g., *M. oleosa* and *M. undata* make one such cluster), or to the contrary, they attributed separate status of non-clustering species to *M. peduncularis*, *M. acuminata*, and *M. cordata*.

The authors of [9] provided another example of the TLC-based chemotaxonomic approach to eight specimens of plants from the family of Lauraceae belonging to two genera, *Cinnamomum* and *Litsea*. The investigation set included four representatives of the *Cinnamomum* genus (*C. bejolghota*, *C. camphora*, *C. tamala* and *C. verum*) and four representatives of the *Litsea* genus (*L. assamica*, *L. glutinosa*, *L. laeta* and *L. monopetala*). The habitat of these plants is in the sub-Himalayan Terai and Duars regions of North Bengal, and the bark extracts of these plants have many medicinal uses which include antioxidant, anti-microbial and anti-inflammatory ones. The reason for the TLC-based research was that the indicated members of the Lauraceae family show variations and similarities in different morphological aspects which pose certain problems with identification of these plants, so that they have been earlier many times re-classified, but with no satisfactory effect.

In the reported experiment, bark samples were collected from all plants and extracted with methanol to obtain secondary metabolites, and they were also hydrodistilled for essential oils. The TLC analyses of the methanol extracts were carried out on the silica gel pre-coated chromatographic plates, using separate solvent systems for different groups of secondary metabolites. For quantification of anthraquinones, ethyl acetate–methanol–water (100:13.5:10, *v*/*v*/*v*) was used, and visualization of chromatograms was carried out at 365 nm in UV light. For quantification of flavonoids and phenolics, ethyl acetate–formic acid–acetic acid–water (100:11:11:27, *v*/*v*/*v*/*v*) was employed, and visualization was carried out at 365 nm in UV light (for flavonoids) and in visible light (for phenolics). Moreover, chromatograms of flavonoids and phenolics underwent the DPPH• test for antioxidant potential of individual fractions. Last but not least, TLC was also used to fingerprint essential oils, and then visualization of chromatograms was carried out by spraying the plates with anisaldehyde followed by heating, while detection was carried out in visible light. Based on multiple test repetitions and high amounts of the collected fingerprint results, cluster analysis was eventually applied to analyse the obtained data, which confirmed chemotaxonomic correlation of the two genera (*Cinnamomum* and *Litsea*) of the Lauraceae family, and the *Cinnamonum* genus proved distinctly separate from the *Litsea* genus. It was also shown that *C. camphora* makes an independent entity, while a close relationship is observed among *C. tamala*, *C. verum* and *C. bejolghota*. In the case of *Litsea*, four genera were divided into two dendrogram branches, one branch representing *L. laeta* and *L. glutinosa*, and the other branch standing for *L. monopetala* and *L. assamica*.

The authors of [22] provided a comparison of TLC fingerprints for the popular medicinal and culinary herbs from the Mediterranean region, which all belong to the Lamiaceae family yet to the three different Lamiaceae genera, *Salvia*, *Dracocephalum* and *Thymus* (and from each individual genus, two different species were selected). Thus, the following six kinds of plants were examined, *S. triloba*, *S. staminea*, *D. moldavica* (variety with white flowers), *D. moldavica* (variety with blue flowers), *T. vulgaris*, and *T. serpyllum*. All six plant kinds underwent selective multistep liquid extraction for phenolic acids and flavonoids, following procedures given in [23], and the detailed protocol was elaborated based on an additional information derived from the literature [24,25,26,27]. Although the protocol of selective multistep liquid extraction of botanicals for phenolic acids and flavonoids has been elaborated a considerable time ago, we regard it as simple and at the same time very effective, and therefore worth recalling and advertising in this review. Thus, the plant extracts were divided into six fractions of phenolic compounds, i.e., the (i) free phenolic acids, (ii) bonded phenolic acids liberated through acidic hydrolysis, (iii) bonded phenolic acids liberated through basic hydrolysis, (iv) flavonoid aglycones, (v) low-polar flavonoid glycosides, and (vi) polar flavonoid glycosides.

An aim of this study was to compare six fingerprints (i)–(vi) for each of the six aforementioned kinds of plants, in order to find out if these fingerprints could allow for distinguishing among the genera of these closely related species. It was found out that chromatographic fingerprints of fraction (iv), i.e., flavonoid aglycons, were the only ones which permitted for correctly ascribing the investigated species to the *Salvia*, *Dracocephalum* and *Thymus* genera, despite the lowest intensity of the respective signals on the chromatograms. Thus, the chromatograms of flavonoid aglycones proved their chemotaxonomic importance as marker fingerprints for individual plant genera.

The TLC method for chemotaxonomic differentiation between two medicinal plants listed in Chinese Pharmacopoeia, i.e., field thistle (*Cirsium setosum*) and Japanese field thistle (*Cirsium japonicum*) was introduced in [28]. Both plants belong to the family of Compositae, and they are important components of traditional Chinese medicines, internally and externally used to treat diverse kinds of bleeding and inflammation. They are herbaceous perennials, and the aerial parts of both plants, which are used medicinally, are difficult to distinguish morphologically, while differentiation of the dried and cut crude plants is even more challenging. A simple TLC method proposed in [28] permits for an unambiguous differentiation, however, of *C. japonicum* and *C. setosum* by their flavonoid fingerprints. To this effect, plant material was extracted with methanol, and then, the chromatograms of the extracts were developed with use of the silica gel pre-coated aluminium sheets and ethyl acetate–formic acid–acetic acid–water (12:1.5:1.5:4, *v*/*v*/*v*/*v*) as the mobile phase. Two characteristic flavonoids, i.e., pectolinarin and linarin, were used as chemotaxonomic markers and external standards for analysis. Visualization was carried out by spraying the plates with the natural products spray reagent (a 1% solution of 2-aminoethyldiphenylborinate in methanol), and after gentle heating, the plates were inspected in UV light at 366 nm for fluorescent spots of linarin (yellow) and pectolinarin (brown). This procedure allowed for distinguishing between *C. setosum* (containing in its extract linarin only) and *C. japonicum* (containing both pectolinarin and linarin).

## 3. Thin-Layer Chromatography Coupled with Bioassays

Thin-layer chromatography hyphenated with a number of bioassays is an excellent strategy for rapid screening of various different botanicals (and in the first instance, medicinal plants and culinary herbs and spices), mainly for their free radical scavenging activity and antimicrobial- and enzyme-inhibiting properties [14,29,30]. This trend of coupling TLC with a variety of other analytical tools is effective and very promising for the future; hence, it is on a rising tide now and is far from having said its last word. According to newly coined terminology, the discussed strategy is often referred to as TLC-EDA, where EDA holds for the effect-directed analysis. Certain bottlenecks in development of new couplings (or hyphenations) are due to technical demands caused by increasingly more sophisticated analytical tools, but inventiveness of researchers is hard to overestimate, and numbers of new technical solutions are steadily growing. In this section, we present a selection of illustrative and practical enough TLC-EDA examples in the three main application fields, focused on (i) the free radical scavenging properties, (ii) the antimicrobial properties, and (iii) the enzyme-inhibiting properties of selected fractions of secondary plant metabolites.

### 3.1. Thin-Layer Chromatography in Screening of Botanicals for Their Free Radical Scavenging Activity

Polyphenolic compounds are commonly found in many herbs (but also in selected fruits, vegetables and even in grain products), and they have been reported to have multiple biological effects, including strong antioxidant activity. There are also other classes of chemical compounds with well-pronounced antioxidant activity, e.g., carotenoids, tocopherols and ascorbates. Currently, there is a growing interest in correlating phytochemical constituents of indigenous plants originating from all different regions of the world with their pro-health antioxidant activity. Methods to determine total antioxidant activity (TAA) of plants are generally based on inhibition of certain reactions by antioxidants present in plant samples. The most widely used methods are those which involve generation of radical compounds which then disappear in the presence of antioxidants. The two most common reference systems employ either 2,20-azino-bis-3-ethylbenzthiazoline-6-sulphonic acid (ABTS) (in the presence of sodium persulfate giving the free radical cation), or the stable 1,1-diphenyl-2-picrylhydrazyl (DPPH•) radical as the reference free radical models, and trolox, gallic acid (GA), or ascorbic acid (AA) as the reference free radical scavenger models, against which set the antioxidant potential of the plant extracts to be calibrated. These (and some other) free radical vs. free radical scavenger interactions produce colour effects, so that quantification of the antioxidant potential is usually performed by means of the UV–VIS spectrophotometric method. Measurement of the antioxidant potential with use of DPPH• as a free radical and trolox, GA, or AA as a free radical scavenger can also be performed with use of the electron paramagnetic resonance (EPR) spectroscopic approach. Although the results originating from the UV–VIS spectrophotometry or the EPR spectroscopy are quantitative and therefore considerably more accurate than the qualitative or semi-quantitative assessments originating from the TLC-EDA tests, they need instrumental equipment (which in the case of the EPR spectroscopy is particularly expensive) and moreover not always available in laboratories engaged in rapid screening of botanicals. Faced with a huge number of plants which have not yet been tested for their antioxidant potential, rapid qualitative or semi-qualitative screening of their extracts by means of TLC hyphenated with the DPPH• test seems to be a very convenient step in the initial diagnosis of plant material for its antioxidant capacity. This is the simplest method, wherein the plant extract is brought into contact with the DPPH• solution and the result is recorded after a certain time. In [31], the authors presented the genesis and development of the DPPH• method, including the basic mechanism which stands behind it. This mechanism can be given in the following manner: If we represent the DPPH• radical by Z• and the free radical scavenging molecule (e.g., phenolic acid) by AH, then the primary reaction is given below:

Z• + AH ⇔ ZH + A•(1)

As a result of this reaction, the deep violet colour of the DPPH• radical disappears and instead, the pale yellow colour of the reduced ZH form is observed. The DPPH• method was first introduced to TLC in 1967 [32] for qualitative assessment of chromatographic plates with the plant extracts developed and fractionated on them. Upon spraying the developed and dried plates with the DPPH• solution, the pale yellow zones appeared on the deep violet background of these plant extract fractions which were endowed with the free radical scavenging potential. In 2005, the reversed phase TLC method combined with the video scanning detection was first developed for quantitative evaluation of the free radical scavenging activity of antioxidative fractions from rapeseed meal by the DPPH• method [33]. Comparison of the results obtained by this approach showed good correlation between the activities measured by TLC-DPPH• and the conventional spectrophotometric assay.

An important paper on optimization of the TLC conditions when studying the free radical scavenging properties of medicinal and culinary herbs was published in 2012 [34]. The authors admitted that although the TLC-DPPH• test belongs to the arsenal of popular and frequently used TLC-EDA strategies with an aim to reveal radical scavenging properties of plant extracts, it has to be employed following certain rules which might allow for a comparison of the data derived from different laboratories. To this effect, the authors focused on selection of a stationary phase most suitable for the TLC-DPPH• tests, and for this purpose, they performed a pilot dot-blot comparison among plain silica gel stationary phase and the chemically bonded stationary phases (NP-CN and RP-18), using for the experiment a wide spectrum of the test compounds abundantly present in botanicals and known for their considerable free radical scavenging potential (e.g., rosmarinic acid, cinnamic acid, caffeic acid etc.). The most active adsorbent (silica gel) unnecessarily strengthens the result of the radical–antioxidant reaction, and the polar bonded stationary phase (NP-CN) unnecessarily weakens it. Based on this observation, it was concluded that (i) the TLC-DPPH• assay should preferably be performed on the surface of a non-specific adsorbent (e.g., RP-18), (ii) the DPPH• reagent should be dissolved in *n*-hexane, and (iii) documentation of results should be made every 5 minutes after staining with the DPPH• solution, as visual effects perceptibly change in the course of time.

Application of TLC to monitor the free radical scavenging activity of nineteen *Salvia* species which were cultivated in Poland and to develop respective free radical scavenging fingerprints of these plants was presented in [35]. Chromatography was performed on the silica gel layers with use of two eluents, one for resolution of the less polar compounds (toluene-ethyl acetate-formic acid (60:40:1, *v*/*v*/*v*)), and the other one for resolution of the medium and highly polar ones (ethyl acetate–water–formic acid–acetic acid (100:26:11:11, *v*/*v*/*v*/*v*)). As reference compounds, the authors used the gallic acid, hiperoside, rutin, caffeic acid, chlorogenic acid and rosmarinic acid standards. Developed plates were sprayed with the vanillin–sulphuric acid reagent (to produce chemical fingerprints) and with DPPH• solution (to generate the free radical scavenging fingerprints). With four *Salvia* species, it was revealed that their strong free radical scavenging properties were not only due to polar flavonoids and phenolic acids present in the extracts, but also due to the other free radical scavengers in the less polar fractions. It was also established that due to similarities in chromatographic and free radical scavenging fingerprints of *S. triloba* and *S. officinalis*, the former one can be regarded as a pharmacopoeial species candidate. The developed method was validated for its specificity, precision (repeatability and intermediate precision), stability and robustness, according to the recognised AOAC guidelines for the qualitative TLC procedures [36].

The authors of [37] provided a report on thirty-six herbal species from the Lamiaceae family belonging to the two plant genera (*Salvia* and *Thymus*) which underwent a multistep extraction described elsewhere [23,24,25]. Six fractions derived from each plant underwent the TLC-DPPH• test to reveal these plants and plant fractions with the most strongly pronounced antioxidant properties, and caffeic acid was selected as an external standard to monitor these properties. It was shown that caffeic acid most abundantly appeared in the fractions derived from all the investigated herbs in the course of basic hydrolysis, but it never appeared in the fractions derived from the acidic one. Moreover, it was once again established that all the analysed fractions of *S. officinalis* and *S. triloba* demonstrate similar free radical scavenging activity, which might serve as a starting point for further studies and eventual proclamation of *S. triloba* as a medicinal plant in its full right, hence deserving its monograph in herbal pharmacopoeia.

In [38], the authors presented the HPTLC-EDA approach to assess the biological properties (the antioxidant and antidiabetic potential) of the ethanol and ethyl acetate extracts derived from ten macroalgae species (three Chlorophyta, four Phaeophyta and three Rhodophyta) originating from the Blue Lagoon beach in Malaysia. Chromatograms were developed with the use of the HPTLC quality silica gel as stationary phase and *n*-hexane–ethyl acetate–acetic acid (20:9:1, *v*/*v*/*v*) as the mobile phase. The experiments were carefully performed, and the method was validated for the contents of the standards employed. A comparison was made of antioxidant potential depending on the extractant used, and it was established that on average, higher antioxidant activity was observed with the ethyl acetate extracts, although the phenolic content was higher in the ethanol extracts. Although the authors did not comment on this observation, it seems justified to suppose that in the case of the discussed macroalgae, not only the phenolic compounds, but also those which are less polar, characterize with well-pronounced antioxidant properties (a similar conclusion was inferred in [35]).

The authors of [39] provided an up-to-the-date review of a selection of the most important studies on natural antioxidants in foods (including beverages), food ingredients, and dietary supplements, performed with the aid of the TLC-DPPH• test (and of TLC hyphenated with some other bioautographic techniques). In one way or another, all examples collected in this review refer to botanicals and to their antioxidant potential, and the review provides a considerable amount of the 73 references from the original research papers.

### 3.2. Thin-Layer Chromatography in Screening of Botanicals for Their Antimicrobial Properties

**Scheme 1:**
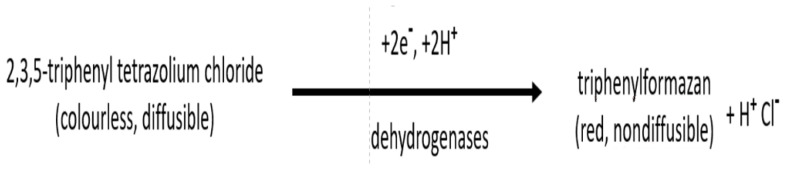
Conversion of colourless tetrazolium chloride to red formazan in the reaction with dehydrogenases of living cells.

In the 1960s, when the “golden era” in drug discovery has come to its end and almost all groups of important antibiotics (tetracyclines, cephalosporins, aminoglycosides and macrolides) were already discovered, it seemed to many that all the main problems of chemotherapy were once and for all solved. Currently, an exciting potential of antibiotics from that “golden era” is in a serious danger of losing its efficacy and importance due to growing microbial multidrug resistance. For this reason, the discovery of new antibiotics is an important objective for public health, and in this context, plants which can provide a huge range of complex and structurally diverse compounds still remain one of major sources of the new drug molecules or molecular concepts for tomorrow. To this effect, many researchers have currently focused on the investigation of plant extracts for their antibacterial activity. A variety of laboratory methods can be used to the preliminary in vitro screening of antimicrobial activity with plant extracts, and the leading traditional approaches are the agar disk-diffusion and the broth or agar dilution methods. All of them are tedious, time-consuming, relatively complicated and needing adequate facilities, and moreover, they demand from the experimenters considerable skills in carrying out these tests. To the contrary, thin-layer chromatography in screening anti-microbial capacity of plant extracts is a far simpler and easier to perform alternative approach, and it can be put into practice in one of the three variants, known as (i) agar diffusion, (ii) direct bioautography, and (iii) agar overlay bioassay. Direct bioautography (DB) is the most often applied method among these three, and it consists in dipping into or spraying with a microbial suspension the developed TLC plate. Then, the bioautogram is incubated at a fixed temperature and for a fixed time under humid condition. To visualize the microbial growth directly on the chromatographic plate, tetrazolium salts are frequently used as a spray reagent. These salts undergo conversion by dehydrogenases of the living cells to the corresponding and intensely coloured formazan, as schematically shown in the below Scheme 1: 

Review [40] contains a thorough and inspiring spectrum of different TLC approaches to characterize plants for their biological properties. It focuses on antimicrobial and antifungal assays, enzyme inhibition, antioxidant testing, and free radical scavenging activity, and it comes with 66 references of the original research papers. A similar review taking on a vast range of the TLC approaches to screening botanicals for their different biological properties is also given in [41], which dispenses a solid number of 99 references to the original research papers. Apart from general reviews which attempt to cover an entire spectrum of the TLC applications to screening different biological properties of botanicals, we also have reviews which cover selected kinds of TLC applications, and a good example is paper [42], which focuses on the screening of antimicrobial properties and is implemented with 75 references. In an interesting paper [11] (published in the Journal of Visual Experiments), a step-by-step explanation (implemented with a nice selection of instructive figures) is provided on how to practically perform the TLC-direct bioautography (TLC-DB) test for plant extracts to identify antimicrobial compounds (with direct bioautography, also known as the dot-blot test). As a working example given in [11], the TLC separation of phenolics extracted from the red clover (*Trifolium pratense* cv. Kenland) plant is presented, followed by screening of the separated fractions for their activity against *Clostridium sticklandii*, a hyper ammonia-producing bacterium (HAB) that is native to bovine rumen.

A kind of precursor to the TLC-DB approach is the dot-blot test alone, performed with aid of the thin-layer chromatographic adsorbent, yet without preliminary chromatographic fractionation of the sample considered. In that way, information is derived on an overall antibacterial potential of the plant extract, but without pointing out any specific fraction or individual compound derived from the scrutinized sample. A good practical example of such an approach is given in [43]. The authors applied this test to compare antibacterial activity of 18 thyme (*Thymus*) specimens and species (originating from the same gardening plot and harvested in the same period). To this effect, polar fractions of the secondary metabolites were derived from each thyme plant, which were then drop-wise deposited on the silica gel pre-coated chromatographic plates, yet without developing the chromatograms. Then, the well-described dot-blot procedure was performed for antibacterial activity against the Gram-positive *Bacillus subtilis* strain. It was established that all investigated extracts exhibited antibacterial activity, yet distinct differences in the size of the bacterial growth inhibition zones were observed among the compared thyme species. Based on the results obtained, *T. citriodorus* “golden dwarf” and *T. marschallianus* were selected as prominent targets for further investigations and possible inclusion in herbal pharmacopeia, which was an essential scientific novelty of this study.

Practical illustration of the TLC-DB assay is provided in [44], and it focuses on two medicinal plants belonging to the European ethnopharmacy, i.e., on *Matricaria recutita* L. (chamomile) and *Achillea millefolium* L. (yarrow). To this effect, tinctures from aerial flowering parts of these plants were prepared by seven days of maceration in 70% ethanol (according to Polish Pharmacopoeia VI) and then chromatographically developed, and the chromatograms with separated fractions were tested against eight bacterial strains, i.e., *Staphylococcus epidermidis*, *S. aureus*, the methicillin-resistant *S. aureus*, *Escherichia coli*, *Pseudomonas syringae* pv. *maculicola*, *Xanthomonas campestis* pv. v*esicatoria*, *Aliivibrio fischeri*, and *Bacillus subtilis*. As a result, considerable antibacterial properties were for the first time confirmed with two compounds found in the examined tinctures, i.e., with apigenin and *α*-linolenic acid, and their identity was additionally confirmed by means of LC/MS.

Rapid screening of botanicals for their antimicrobial properties is advantageous, especially with plants originating from sub-tropical and tropical regions which are more abundant in flora and which are relatively less researched than flora originating from the temperate climatic zones. In that way, a shortcut verification can be assured of healing potential with traditional local medicines, which in terms of accessibility and use are ahead of Western medicines. A working example is provided in [45], which focuses on antibacterial properties of the leaf extract derived from the Philippine *Piper betle* L. plant belonging to the family of Piperaceae, which is recognized in India, Sri Lanka, Malaysia, Philippines and the other subtropical countries for its antibacterial, cytotoxic, hepatoprotective and many other advantageous pro-health properties. From the research data obtained from instrumental techniques (more advanced than the TLC-DB assay), it has already been known that the methanol, ethanol and supercritical CO_2_ leaf extracts from *P. betle* are exceptionally active against a number of the multidrug resistant (MDR) bacteria. In the discussed study, the ethanol leaf extract of *P. betle* was separated with use of TLC into eight fractions, which then underwent the dot-blot test. To this effect, the TLC system used consisted of the silica gel stationary phase and ethyl acetate–*n*-hexane (7:3, *v*/*v*) mobile phase. Two spots with *R*_F_ values of 0.86 and 0.13 showed inhibitory activities against two Gram-positive MDR bacteria, i.e., the methicillin-resistant *Staphylococcus aureus* and the vancomycin-resistant *Enterococcus*. The spot with *R*_F_ = 0.86 also showed inhibitory activity against two Gram-negative MDR bacteria, i.e., the carbapenem-resistant Enterobacteriaceae, *Klebsiella pneumoniae* and the metallo-𝛽-lactamase-producing *Acinetobacter baumannii*. With aid of the GC/MS technique, six compounds contained in the spots showing antibacterial activity were identified, with four of them never before having been mentioned in the medical literature.

In [12], the authors present the most up-to-the-date overview of the TLC-DB application to phytochemistry, and its two considerable advantages are that it provides a chronological order of development of this technique (nicely combined with dynamic development of its application range) and that it is largely focused on applications of TLC to scrutinize traditional Chinese medicines (TCM), to which it is the best placed. What sets this most recently published review apart from the others is that it provides preliminary information on attempts to identify natural products with an anti-COVID potential inherent of medicinal plants. It points to [46], which connects plant material with its possible efficiency in alleviating and/or combating the COVID-19 risks (which in the first instance are respiratory syndromes). Namely, in [46], a report is given on the in silico study of eleven Indian herbal plants with putative inhibitory properties against COVID-19. From this study, it comes out that components of two plants, *Nyctanthes arbortristis* (harsingar) and *Aloe barbadensis* Miller (*Aloe vera*), are the most promising ones which might display anti-COVID-19 potential. For this reason, these two plants should be selected for future steps of the experimental studies.

### 3.3. Thin-Layer Chromatography in Screening of Botanicals for Their Enzyme-Inhibiting Potential

Many drugs are inhibitors of enzymes involved in mediating disease processes, and the same can be said about numerous plant constituents. Understanding the mechanism of action (MOA) of the target enzyme is critical in early discovery and development of drug candidates through extensive structure–activity relationship (SAR) studies. The purpose of a mechanism of action (MOA) study is to characterize the interaction of a compound with its target enzyme to understand how the compound interacts with this target and how natural substrates at physiologic concentrations can modulate this activity. These compounds which prove as inhibitors of enzymes rarely become proper drugs, however, due to strict requirements for a drug not only to inhibit the target but to have acceptable solubility, permeability, protein binding, and the selectivity, metabolism and toxicity profiles. This potential for the compound to become a proper drug is slowly revealed through tracking these characteristics in the specially designed structure–activity relationship (SAR) studies.

Similar to what has been stated in the preceding section, plants can offer a huge range of complex and structurally diverse compounds, and for this reason, they still remain one of the major reservoirs of the new drug molecules or molecular concepts for tomorrow, also in their capacity as enzyme inhibitors. Rapidity and cost-effectiveness of screening plant extracts with an aid of TLC-direct bioautography and human or mammal enzymes for testing their enzyme inhibiting capacity is a very attractive option for medicinal chemists in their search for novel structural motifs. To this effect, plant extract undergoes thin-layer chromatographic separation to individual constituents and/or constituent fractions, and then the dried chromatographic plate is sprayed with enzyme solution followed by a strict incubation protocol under humid conditions, and finally, it is visualized by spraying the plate with a visualizing reagent (which in the case of tracing cholinesterase or glucosidase inhibitors, is often the Fast Blue B salt).

Currently, one of the most acute health conditions among ageing populations worldwide is Alzheimer’s disease, and for this reason, the inhibitors of acetylcholinesterase (AChE) and butyrylcholinesterase (BuChE) currently form the basis of the newest drugs available for management of this disease. In view of a considerable potential of plants, the TLC bioautographic assays have been performed for screening plant extracts in the search for natural cholinesterase inhibitors. Review on these inhibitors which are available from natural botanical sources [47], implemented with 76 references to the original research papers, introduces a long list of plants recognized for their cholinesterase-inhibiting activity. These plants have long been used in ethnomedicines, particularly for memory-related disorders, and their efficiency is attributed to specific alkaloids, terpenes, sterols, flavanoids and glycosides. The most potent cholinesterase inhibitors are observed with the components of plants from the families of Buxaceae, Amaryllidaceae and Lycopodiaceae, although certain activity has also been established with the extracts from plants belonging to the families of Lamiaceae, Chenopodiaceae, Papaveraceae, Apocynaceae, and Labiatae. A number of papers have been published on this subject matter (e.g., [48,49,50,51,52]), and the basic principle of all these methods is that the enzyme (cholinesterase) converts 1-naphthyl acetate into naphthol, which reacts with Fast Blue B salt to make a purple-coloured background on TLC plates. Thus, upon the thin-layer chromatographic fractionation of the plant extract, the plate is dried for complete removal of liquid mobile phase, and then, it is sprayed with the enzyme stock solution. Then, it is again dried and kept in humid atmosphere for incubation at 37 °C for 20 min, and eventually, it is sprayed again with solution of *α*-naphthyl acetate and Fast Blue B salt, to give purple colouration after 1–2 min. The AChE and BuChE enzyme-inhibiting zones (i.e., fractions derived from the plant extracts) produce white spots on the purple background.

Among the first reports on a possibility of screening plant extracts for the detection of the cholinesterase-inhibition zones was that issued by Verpoorte et al. [48], who used acetylcholinesterase inhibitors from the Amaryllidaceae extracts in the TLC-DB test instead of synthetic galanthamine. Working conditions of the TLC-DB assay (which follows a very detailed protocol) were tested upon two alkaloids (galanthamine and physostigmine), and the results were reported in [49]. Although galanthamine and physostigmine are currently produced synthetically and are used for the treatment of cognitive decline in mild to moderate Alzheimer’s disease, both alkaloids have originally been isolated from plants and, more specifically, from the bulbs and plants of *Galanthus nivalis* (known as common snowdrop) and *Physostigma venenosum*, a leguminous plant endemic to tropical Africa. Over time, working conditions of the original TLC-BD method were modified, and in [50], an improved methodology was proposed focusing on the concentration of enzymes, the reagents, and the reaction time. As a consequence, the consumption of enzymes was reduced by 85%, and the detection limits were remarkably decreased. The authors of [51] provided a comparison between the performance of the TLC-DB assay for inhibitors of cholinesterase and the 96-well plate assay based on Ellman’s method. For the majority (83%) of the 138 test compounds of natural and synthetic origin, the results obtained with the two assays have converged, and both screening assays were considered as suitable for the generation of new hits. The TLC-DB methodology for the screening of plant extracts in the search for new botanical inhibitors of cholinesterase is still in use, and in [52], a report is given on the examination of the never-before-tested single components from the ginger (*Zingiber officinale*) extract. The experiment led to recognition of three active inhibitors among volatile constituents of this plant, i.e., *ar*-curcumene, *α*-sesquiphellandrene, and *α*-zingiberene. Identification was possible owing to the TLC-HPLC-MS interface analysis of active zones and the GC-MS analysis of tested samples. The authors of [53] provided another report on the TLC-DB test of the methanol extract of *Schisandra chinensis* for the cholinesterase inhibition effect, and the dibenzocyclooctadiene lignans contained in this plant were proven as responsible for the inhibition.

Along with Alzheimer’s disease, obesity (i.e., an abnormal or excessive fat accumulation) is also considered as one of great threats to human health on a global scale. It tends to aggravate the chances of acquiring diseases such as type 2 diabetes, hypertension, fatty liver, and cancer, which in turn reduce both life expectancy and the quality of life. Pancreatic lipase is a key enzyme for digestion of triacylglycerols, and inhibition of lipase has until now been the most explored strategy for treatment of obesity. In recent years, increasingly more medicinal herbs have been reported to show inhibitory activities against pancreatic lipase, and discovery of lipase inhibitors among herbal medicines may provide potential alternatives for the treatment of obesity. The authors of [54] provided a report on the TLC-DB screening of methanolic extracts from the *Camellia sinensis* (L.) Kuntz, *Rosmarinus officinalis* L. and *Morus alba* leaves, for their ability to inhibit pancreatic lipase. Upon the thin-layer chromatographic fractionation of the plant extracts in the TLC system composed of silica gel as stationary phase and ethyl acetate–methanol–water (60:30:10 *v*/*v*/*v*) mobile phase, chromatographic plates were sprayed with *α*-naphtyl acetate, and the enzyme solutions before incubation at 37 °C for 20 min. Finally, solution of Fast Blue B salt was sprayed onto the TLC plates, giving a purple background colouration. Orlistat (a synthetic lipase inhibitor drug) was used as a reference analyte. The pancreatic lipase inhibiting zones of the plant extracts and the reference drug zone appeared as white spots on the purple background. In that way, it was demonstrated that the extracts from *C. sinensis* and *R. officinalis* exert an inhibitory effect upon pancreatic lipase, whereas *Morus alba* lacks analogical activity.

An alternative TLC-DB procedure was also proposed for screening pancreatic lipase [55], and to this effect, the *p*-nitrophenyl butyrate (PNPB) and bromothymol blue system were used to detect the lipase inhibition fractions derived from three unexplored species of *Streptomyces* (*S. tendae*, *S. aurantiacus* and *S. albaduncus*), with orlistat used as a positive control of procedure performance. Upon developing orlistat and supernatants derived from the *Streptomyces* samples on the silica gel pre-coated chromatographic plates (with chloroform–methanol (90:10, *v*/*v*) for orlistat and benzene–methanol (60:40, *v*/*v*) for *Streptomyces*), the plates were air dried until the mobile phase was evaporated completely. Then, a complex yet well-described procedure involving the porcine pancreatic lipase enzyme was implemented, and finally, the inhibitory zones were visualized as blue spots against the greenish-yellow background. 

The authors of [56] elaborated a thorough report on usage of the TLC-DB test to screening the Chinese medicine Ophiopogonis Radix (which is the dried root of *Ophiopogon japonicus* (L.f) Ker-Gawl.) for its ability to inhibit pancreatic lipase. To this effect, the HPTLC method was used, which employed the two-step gradient elution with two different mobile phases for simultaneous analysis of seven constituents of Ophiopogonis Radix. Direct bioautography was performed for plant samples originating from two different regions of China (i.e., the Sichuan and the Zhejiang province) and/or from different growth years. Visualization of the enzyme-inhibiting effect was performed in a standard manner by spraying the developed and incubated plates with the solution of *α*-naphthyl acetate and Fast Blue B salt. The lipase inhibition essays showed that five compounds contained in the plant extracts (i.e., ophiopogonin D, ophiopojaponin C, ophiopogonin D’, ophiopogonin C’ and methylophiopogonanone B) demonstrated an inhibitory effect, yet its strength largely depended on the plant vegetation region and the growth year. In that way, not only confirmation was obtained of the Ophiopogonis Radix potential as an active lipase inhibitor, but a quality control method was also proposed for this herbaceous material.

Review of the application of the TLC-DB approach as a tool for rapid detection of enzyme inhibitors is given in [57]. A statement is made that the TLC-DB methods have been developed for several classes of enzymes including oxidoreductases, hydrolases and isomerases, and there is a potential for developing functional methods for the other classes of enzymes as well. In [57], the authors provide a summary of the known TLC-DB methods and their applications for the determination of enzyme inhibitors in plant extracts, and a comparison is made of the effectiveness of different methodological approaches, which points to the current state and to the perspective of the development of the TLC-DB methodology used for the screening of botanicals for their enzyme-inhibiting potential and to its possible future applications. Thus, the fast screening of botanicals for natural enzyme inhibitors of plant origin can justifiably be regarded as an inspiring prelude in the search for novel structural motifs in drug development endeavours.

## 4. Thin-Layer Chromatography in Screening of Medicinal and Culinary Herbs and in Quality Control of Alimentary and Cosmetic Products of Botanical Origin

### 4.1. Thin-Layer Chromatography in Screening of Medicinal and Culinary Herbs

There are three main factors which limit rational use of medicinal herbs, and these are uncertainty on their effectivity, uncertainty on their safety and variation in their quality. Uncertainty regarding effectivity of herbal medicines is not an issue which can be tackled with use of the TLC-based methodology, as it remains entirely within the competence of medical sciences. Thin-layer chromatography is an excellent practical tool, however, which allows for issuing binding opinions regarding quality of herbs, as economical motivation of fraud on herbs (either medicinal, or culinary) is widely known, and it can seriously endanger public health. Authentication and quality control of herbal medicines is of a particular importance in all these regions where herbal ethno-medicines play a prevalent role in health care of the population. Similar precautions refer to culinary herbs and spices, which—contrary to ethno-medicines of a local importance only—can also be quite expensive and hence become a temptation for fraudulent actions. Moreover, culinary herbs and spices are abundantly traded worldwide. Quality control of medicinal (and culinary) herbs is very much an essential requirement to maintain the quality of them, yet the regulation norms for herbs are not as strict as when compared to synthetic medicines. Although the World Health Organization and European Union have issued guidelines which define the basic criteria for evaluation of quality, safety, and efficacy of herbal medicines with the goal of assisting national regulatory authorities, there are still different ways in which countries define medicinal herbs or products derived from them. A comprehensive review on TLC in quality control and safety of botanicals is given in [58]. 

An example of an impressive versatility of TLC in quality control of three medicinal herbs recognized by Korean Pharmacopoeia IX, which are radix of *Angelica gigas* Nakai (Umbelliferae) from Korea, fruit of *Evodia rutaecarpa* Bentham (Rutaceae) from China and fruit of *Schisandra chinensis* Baillon (Schisandraceae) from China, is given in [59]. Firstly, herbs were extracted with use of the methods listed in Korean Pharmacopoeia IX, and then, extracts were developed on the silica gel pre-coated plates with use of carefully selected mobile phases. Extract of the *Angelica gigas* radix was developed with hexane–ethyl acetate–methanol (3:2:1, *v*/*v*/*v*), and visualisation of chromatograms was made in UV light at 365 nm. Marker compounds for *Angelica gigas* were decursin and decursinol. Extract of the *Evodia rutaecarpa* fruit was developed with dichloromethane–methanol–formic acid (40:1.5:2, *v*/*v*/*v*), and visualization of chromatograms was made with the ethanolic H_2_SO_4_ in UV light at 365 nm. Marker compounds for *Evodia rutaecarpa* were evodiamine and rutaecarpine. Extract of the *Schisandra chinensis* fruit was developed with toluene–ethyl acetate–formic acid (7:3:0.5, *v*/*v*/*v*), and visualization of chromatograms was made with the ethanolic H_2_SO_4_ in UV light at 365 (for gomisin A and gomisin N) and at 254 nm (for schisandrin). Mass spectra of the spots were registered directly on the chromatograms by placing the developed plates between the DART ion source and the TOF analyser, in the so-called TLC-TOF-DART/MS hyphenation mode (the approach which was first proposed in [60]). The *m/z* signals detected in the mass spectra of marker compounds and then confirmed as present in the mass spectra of the three medicinal herb extracts were an ultimate authentication step for these plants.

The authors of [61] presented a similar example of the quality control of selected herbs belonging to the Chinese medicinal system, yet with use of another hyphenated TLC-MS approach. The authors targeted eight medicinal plants including *Sophorae Flavescentis* Radix, *Angelicae Sinensis* Radix, *Acori Tatarinowii* Rhizoma, *Phellodendri Chinensis* Cortex, *Picrasmae* Ramulus et Folium, *Gynura Japonica*, *Rhei* Radix and Rhizome, and *Dendrobii* Caulis. Botanical material was first extracted, and then, the extracts were developed (along with respective marker compounds) by means of TLC, and an ultimate identification step was performed with use of laser-ablation of the developed chromatographic plates and direct analysis in the real-time mass spectrometry system (the TLC-LA-DART/MS hyphenation mode). Also in this case, taxonomic markers of individual herbs were identified on chromatographic plates in the form of characteristic *m/z* signals, which allowed for ultimate authentication of the herbs under scrutiny. Details of the performed extraction and the TLC analysis are given in [61].

Still, different hyphenations of TLC with one more advanced instrumental detector, i.e., SERS (where SERS holds for the surface-enhanced Raman spectroscopy), used to be applied as the TLC-SERS hyphenation mode for rapid screening of botanical material. The authors of [62] provide an example of the TLC separation combined with the SERS identification of four main *β*-carboline alkaloids (harmalol, harmaline, harmane and harmine), characteristic of the seed extract from Syrian rue (*Peganum harmala*). The plant itself has been recognized in the Mediterranean basin from times immemorial for its well-pronounced medicinal properties in the curation of dull eyesight, and recently, *β*-carbolines have drawn attention of the medical world for their significant antitumor activity. For the sake of experiment, extraction of alkaloids from the *Peganum harmala* seeds was carried out with methanol. Then, the seed extracts and four commercial alkaloid standards were spotted on to the silica gel pre-coated plates, and chromatograms were developed with CHCl_3_-CH_3_OH-10%NH_3_ (80:20:1.5, *v*/*v*/*v*). Small amounts (0.8 and 0.1 μL, respectively) of the Ag colloid and the 0.5 M KNO_3_ solution were dropped upon each spot and were visualized in UV light, and good quality Raman spectra were recorded at four different excitation wavelengths (1064, 785, 633 and 488 nm) while the spots were still wet. Finally, Raman spectra of the four *β*-carboline alkaloid standards were considered as fingerprints, which allowed for easy identification of the same compounds in the seed extract. 

Expensive TLC systems hyphenated with mass spectrometric, or Raman spectroscopic detectors, are often not available in laboratories oriented on fast screening and authentication of botanicals, and such hyphenated systems are especially missing in laboratories of developing countries. However, expensive systems are not always a necessary precondition to perform a quality screening of herbal material, and often simple TLC equipment is enough in this regard. An example of the quality control of popular medicinal and culinary herbs, *Heterotheca inuloides, Citrus aurantium, Peumus boldus, Equisetum arvense, Eucalyptus globulus, Ginkgo biloba, Mentha piperita, Aloe vera, Salvia officinalis* and *Cassia senna* with use of TLC alone is given in [63]. Each plant was extracted with the ethanol–water solution (90:10, *v*/*v*), following a carefully elaborated extraction procedure. For each herbal extract, a separate TLC system was developed, and appropriate standards were procured, which allowed for the development of the calibration curves, establishment of the LOD and LOQ values for each standard and development of a validated authentication and quality control method for each herb considered.

Screening and authentication of medicinal and culinary herbs and spices is a very specific challenge, due to the fact that they are most often traded on an international scale in a fragmented or pulverized form, which facilitates adulteration, usually by partial replacement of a more expensive botanical with a cheaper substitute. A TLC method was presented for the quality control of ground black pepper (*Piper nigrum* L.), which used to undergo deliberate falsifications with cheaper botanical substituents, e.g., with the ground chili pepper [16]. The first step was elaboration of the calibration curve for the piperine standard as a taxonomic marker for black pepper (which is absent from the chili pepper and allows for quick differentiation between the two). Thus, a series of six different piperine aliquots from 0.10 to 0.60 μg piperine spot^−1^ in the 0.10 μg piperine spot^−1^ intervals was deposited on the silica gel pre-coated TLC plates, and acetone–*n*-hexane (3:2, *v*/*v*) was used as the mobile phase. Then, the investigated black pepper sample underwent an exhaustive extraction with dichloromethane at 70 °C, in order to completely remove the piperine and to prepare a blank black pepper matrix. Eventually, the elaborated method was used in the experiment, which consisted in spiking the blank black pepper matrix at four different concentration levels with piperine, followed by a single extraction run and determination of piperine obtained in the extracts from the spiked blank matrix with use of the developed TLC method. Efficiency of the method was positively confirmed upon a number of control experiments.

### 4.2. Quality Control of Alimentary and Cosmetic Products of Botanical Origin

Quality control of alimentary and cosmetic products of botanical origin is a very broad analytical field which covers different issues, such as adulteration with cheaper botanicals and prohibited synthetic dyes, different kinds of contamination, e.g., with heavy metals, pesticides and other agrochemicals, etc. Thin-layer chromatographic methods are abundantly developed for practically each possible application area, and in this section, a few examples are given of a vast number of different practical cases.

The authors of [64] presented the first from-start-to-end TLC method of fingerprinting the *Cistus incanus* L. raw herbal material, with a purpose to further use it for rapid screening, authentication, and quality control of the traded *C. incanus* L. herbal teas. The efficiency of this method was tested upon twelve *C. incanus* L. samples of different origins (Turkey, Albania and Greece) and of unknown vegetation regions and harvesting periods, randomly purchased from a local market. Samples were first extracted by means of the accelerated solvent extraction (ASE) with chemometrically optimized solvent extraction mixture and temperature (methanol–water, 27:73, *v*/*v*; 130 °C), to derive polar fraction from the plant samples. Then, the extracts were developed in the two different thin-layer chromatographic systems, both using the silica gel pre-coated plates but two different mobile phases: (i) ethyl acetate–formic acid–acetic acid–water (100:11:11:13, *v*/*v*/*v*/*v*), and (ii) ethyl acetate–dichloromethane–formic acid–acetic acid–water (100:10:10:10:11, *v*/*v*/*v*/*v*/*v*). The developed and dried chromatograms were densitometrically scanned in the fluorescence mode at the wavelength λ = 366 nm. Visual inspection of both sets of the obtained chromatographic fingerprints (i.e., the densitograms of herbal extracts) confirmed the authenticity of all investigated samples as the *C. incanus* L. species but revealed a considerable difference in terms of contents of polar fraction, regarded as a specific marker of pro-health properties of herbal teas. These differences could be due to a vast number of reasons (e.g., genetic differences among the plants, different environmental and climatic conditions of the herbs growing, different harvesting periods, different drying and storage conditions, etc.).

In [65], the authors described the development of a novel TLC method of authentication of the anthocyanins- and anthocyanidins-containing alimentary products, despite the great vulnerability of these botanical pigments due to their confirmed chemical instability, and especially in contact with silanols of the silica gel type stationary phase. In order to overcome this considerable difficulty and to ensure stability of anthocyanins in the course of analysis, the reversed-phase chromatographic system was developed based on the RP-18 stationary phase (which ensures a protective retention mechanism with the non-localized adsorption on octadecyl ligands), acetic acid as the mobile phase component and triple development of chromatograms. Two anthocyanins (cyanin and keracyanin) and two anthocyanidins (pelargonidin and delphinidin) were used as phytochemical standards. The first development was carried out with mobile phase I (acetonitrile–methanol–glacial acetic acid (16:4:0.15, *v*/*v*/*v*)) to the distance of 90 mm from the lower plate edge. The second development was carried out with mobile phase II (methanol–glacial acetic acid (20:0.15, *v*/*v*)) to the distance of 70 mm from the lower plate edge. The third development was carried out with mobile phase III (methanol–glacial acetic acid (20:0.45, *v*/*v*)) to the distance of 60 mm from the lower plate edge. For cyanin and keracyanin, densitometry was performed in the absorbance mode at the wavelength 545 nm, for pelargonidin at the wavelength 450 nm, and for delphinidin at the wavelength 555 nm. The developed and validated method was successfully used to identify and quantify cyanin, keracyanin, pelargonidin and delphinidin in selected alimentary products (syrups, juices and herbal infusions).

A novel and validated TLC method was presented for the analysis of two isomeric biphenyl neolignans, magnolol and honokiol, derived from the *Magnolia officinalis* bark and regarded as traditional Oriental medicines [66]. Currently, the *Magnolia* bark extracts and powders are abundantly added to a variety of pro-health dietary supplements traded in the form of tablets, capsules and liquids. To this effect, the magnolol and honokiol standards were prepared in methanol, and silica gel and *n*-hexane–ethyl acetate–ethanol (16:3:1. *v*/*v*/*v*) were used, respectively, as stationary and mobile phase. Densitometric scans of chromatograms were performed in absorbance mode, at the wavelength 290 nm. Based on the developed calibration curves for each individual neolignan, the contents of magnolol and honokiol were assessed in six dietary supplements from a local market (four supplements had a label declaration of the neolignans quantity per one table/capsule/vial, and two supplements missed any declaration). In two preparations, neither magnolol nor honokiol was detected, either due to their absence in these two samples, or because of their contents falling below the LOD values established for the method. Thus, the developed TLC method was shown as an efficient and reliable tool for quantitative determination of the two magnolia neolignans, magnolol and honokiol, in dietary supplements. The obtained quantitative results once again clearly suggest a necessity of strict quality control of dietary supplements to exclude marketed products with doubtful constitution and hence doubtful physiological effects.

Two examples of the TLC application to quality control of cosmetic raw materials are given in [67,68]. Recently, growing interest has been observed in preventive and “anti-ageing” medicine, and for this reason, great attention has been attracted to *trans*-resveratrol known for its excellent antioxidant properties and hence as an excellent additive to cosmetic preparations. For economic reasons, the main sources of *trans*-resveratrol for the cosmetic industry are plants and plant extracts, and the most popular plant is the common grape vine (*Vitis vinifera* L.). The authors of [67] proposed a simple and validated TLC method to determine *trans*-resveratrol in such botanical raw materials with wide application in modern cosmetics, as red wine, dry red wine, extract from red wine, extract from skin of red grapes, extract from the American blueberry juice, extract from fruit wine used in “wine spas”, etc. The TLC method was developed in the reversed phase mode with the RP-18 pre-coated plates and methanol–water (6:4, *v*/*v*) mobile phase. Densitometric detection was carried out in the fluorescence mode (the irradiation wavelength λ = 340 nm), which enabled quantification of *trans*-resveratrol in the cosmetic raw materials of natural origin. The presence of *trans*-resveratrol in the analysed samples was additionally confirmed by visualization of chromatograms with anisaldehyde as a selective visualizing agent.

Two thin-layer chromatographic methods were proposed to facilitate detection of two anthocyanins (cyanin and keracyanin) and two anthocyanidins (pelargonidin and delphinidin) in a selection of homemade and commercial fruit juices, and in infusions prepared of the dried plants, with an applicability to cosmetics [68]. As stationary phase, microcrystalline cellulose was used in the form of the commercially pre-coated chromatographic plates. For the detection of pelargonidin in the investigated juices and infusions, conc. hydrochloric acid–80% formic acid–water (9:46:90, *v*/*v*/*v*) was proposed. For the detection of the remaining three pigments (cyanin, keracyanin and delphinidin) in the analogical samples, 80% formic acid–water–*n*-butanol (16:19:65, *v*/*v*/*v*) was used. Upon drying, the chromatograms were densitometrically scanned, and they were assessed in daylight, UV light (at 254 nm), and upon visualization in the ammonia vapours (in a basic environment, the investigated plant pigments change their colour from pink or red to blue or navy blue). Fifteen products with alimentary and cosmetic applications were tested for the contents of the discussed plant pigments, and among them, the home-made raspberry, blueberry, chokeberry and elderberry juices, analogical products from local discounts and pharmacies, home-made infusions of dried blueberries, hibiscus flower, etc.

## 5. Planar Chromatography in Screening of Psychoactive Plants

Psychoactive plants and fungi have been with humankind since prehistoric times. Some anthropologists even suggest that these organisms might have played an evolutionary role in the mental development of humans [69]. There is firm evidence that they have been included in spiritual practices and ancient rituals of many different cultures worldwide. Some of these practices have survived until our time (e.g., among the tribes of the South American and Mesoamerican Indians), thus enabling diverse anthropologic and medical surveys of this phenomenon [70,71]. Two fungi that are particularly popular for their hallucinogenic properties in the aforementioned regions are the psilocybin mushroom (also known as the psychedelic or “magic” mushroom, *Psilocybe semilanceata*) [72] and sage of the diviners (*Salvia divinorum*) [73]. In many different parts of the Old World, consumption of psychoactive plants also took place, as documented over the centuries since the Neolithic era [74]. Currently, in modern societies worldwide, a need for easily available recreational substances has developed in an effort to temporarily alleviate growing challenges and tensions of an everyday life. An intensified intercontinental tourism enhanced this trend by bringing together tourists from the better-off regions with whole populations in developing countries who make recreational use of local herbs known for containing psychoactive components (e.g., the marijuana-containing cannabis plant in the Indian subcontinent and most of the South Asian region, the cocaine-containing coca leaves in Latin America, the amphetamine-like cathinone contained in the khat leaves from the Arabian Peninsula, the Horn of Africa, etc.) As a result, a burning need has emerged for rapid and efficient screening of psychoactive plants, and an interesting review on this subject matter is provided in [75]. The authors of this paper have focused on the screening methods for psychoactive plants with use of a variety of analytical techniques, including TLC. In this section, we present selected examples of the paper and thin-layer chromatographic applications to screening psychoactive plants and fungi for their most characteristic ingredients.

*Argyreia nervosa* (from the family of Convolvulaceae, known under a number of different common names such as Hawaiian baby wood rose) is a perennial climbing vine native to the Indian subcontinent and with time introduced to many places worldwide including Hawaii, Africa, and the Caribbean. Powerful psychoactive properties of the Hawaiian baby wood rose are due to the ergoline alkaloids contained in its seeds. Two reports have been released on applications of the preparative layer chromatography and paper chromatography to analyse ergoline alkaloids contained in this plant. The first report originates from as early as 1965 [76]. Following the alkaloids-targeting liquid extraction of plant seeds, a fraction of the ergoline alkaloids was isolated by preparative layer chromatography. Eventually, these compounds were separated by means of paper chromatography, using butanol–acetic acid–water (4:1:1, *v*/*v*/*v*) as the mobile phase. It was established that the content of ergoline alkaloids in the Hawaiian baby wood rose seeds equals ca. 3 mg per gram of seeds, with one eighth of this amount being lysergamide. The second report on the analysis of ergoline alkaloids contained in the same plant by means of the preparative layer chromatography followed by paper chromatography comes from 1973 [77]. In this case, nineteen indole alkaloids were identified by the thin-layer and paper chromatographic procedures. Lysergene, festuclavine, setoclavine, isosetoclavine, agroclavine, elymoclavine, ergine, and isoergine were first isolated by means of the column chromatography and were then characterized by TLC and IR. Penniclavine, chanoclavine-I, chanoclavine-II, ergometrine, ergometrinine, lysergic acid α-hydroxyethylamide, isolysergol, racemic chanoclavine-II, molliclavine, lysergol, and isolysergic acid α-hydroxyethylamide were identified by TLC alone. 

*Catha edulis* (from the family of Celastraceae, known under the common name of khat, or qat) is a flowering plant native to Ethiopia and the Horn of Africa. Khat contains the alkaloid cathinone, a stimulant which is known to cause excitement and euphoria upon chewing its evergreen leaves. Because of a significant psychotropic potential of khat (widely used as “natural amphetamine”), the demand for a specific, sensitive and rapid method for determination of its psychoactive principle, the monoamine alkaloid S-(-)-cathinone, in the plant material resulted in elaboration of an efficient TLC screening method [78]. For this purpose, the plant sample was extracted with methanol–0.1 N HC1 (90:10, *v*/*v*), and the TLC analysis was performed with use of the silica gel pre-coated HPTLC plates and ethyl acetate–methanol–ammonia (25%) (85:10:5, *v*/*v*/*v*) as the mobile phase. Densitometric scans of the chromatograms were performed in the absorption/reflectance mode, at the wavelength 205 nm.

A review paper on TLC in the analysis of the hemp plants (commonly known as cannabis, belonging to the family of Cannabaceae, and having three recognized genera, *Cannabis sativa*, *Cannabis indica*, and *Cannabis ruderalis*), on their chemical composition and synthetic cannabinoids contains 84 references [79]. This paper focuses on the most important examples of the analysis of the cannabis variants and their components and on the analysis of synthetic cannabinoids related to their medical and recreational uses. It is known that the cannabis plants have been recognized for their medicinal properties for thousands of years now. Over 700 varieties of cannabis that contain hundreds of compounds are currently known, which include fatty cannabinoids that are the main biologically active constituents and volatile terpenes that have distinct odours. The most important component of all cannabis genera is tetrahydrocannabinol (THC), which provides euphoric effects and makes them popular for use as recreational drugs, alternative medicines, and clinical research drugs. Although the TLC methods cited in this review paper complement more difficult to perform and more expensive HPLC and GC methods (basically the HPLC/MS and GC/MS ones), the TLC methods alone are also used, and they are especially valuable and are often sufficient for the separation, detection and identification of cannabinoids in resources-limited countries. Valuable information in this respect is contained on the website of the HPTLC Association [80] and it contains examples of the NP and RP separations of cannabinoids which allow for their fingerprinting and identification. For the needs of the NP HPTLC analysis, the cannabis plant is extracted by sonication with the methanol–hexane (9:1, *v*/*v*) mixture, and for the needs of the RP HPTLC analysis, it is extracted with pure methanol. The recommended NP HPTLC analysis ought to be performed on the silica gel stationary phase with *n*-heptane–diethyl ether–formic acid (75:25:0.3, *v*/*v*/*v*) as the mobile phase. Visualization is recommended by the spray or dip derivatization with solution of the Fast Blue salt. The recommended RP HPTLC analysis ought to be performed on the RP-18 stationary phase with methanol–water–acetic acid (70:15:15, *v*/*v*/*v*) as the mobile phase.

*Pausinystalia johimbe* (from the family of Rubiaceae) is native to the tropical West Africa and is widely grown in Cameroon, with yohimbine being the major psychoactive alkaloid present in the bark of this plant. Upon extraction of the stem bark of *P. johimbe* with methanol, HPTLC was successfully employed for quantification of yohimbine [81]. For this purpose, the chromatographic system comprised the silica gel pre-coated HPTLC plates and toluene–methyl acetate–diethyl amine (7:2:1, *v*/*v*/*v*) as the mobile phase. Quantification was performed by densitometric scanning of the chromatograms at 285 nm in the reflectance-absorbance mode. The response to yohimbine was linear over the concentration range of 400 to 1200 ng per band, and the method was validated for selectivity, linearity, accuracy, recovery, precision, and robustness.

*Piper methysticum* (from the family of Piperaceae, known under the common name of kava or kava kava) is a crop of the West Pacific Islands (Hawaii, Samoa, Fiji, Pohnpei, etc.). The root of this plant is used to produce a drink with sedative, anaesthetic and euphoriant properties, largely consumed on festive occasions and regarded as an identity symbol of populations of the South Pacific Islands. Active ingredients of kava called kavalactones are known for various psychotropic effects, including the anxiolytic, sedative, and hypnotic action. Kavalactones and flavokavins are the most characteristic components of kava, and they are used as targets for rapid TLC screening of the kava raw material. The HPTLC detection and quantification method for routine assessment of flavokavins contained in the cultivars of kava is given in [82]. Upon extraction of the dried and pulverized plant material with acetone, the HPTLC system used for separation of the extract components includes the silica gel F_254_ pre-coated HPTLC plates and hexane–dioxane (8:2, *v*/*v*) as the mobile phase. Visual inspection and documentation of the chromatograms is carried out at 254 and 366 nm. Densitometric quantification is carried out in the reflectance mode at 366 nm. In [83], the HPTLC identification and quantification method applied to nine compounds from the groups of kavalactones and flavokavins was proposed, based on the same HPTLC system as that introduced in [82].

*Salvia divinorum* (“sage of the diviners”) from the family of Lamiaceae, known under a number of common names such as seer’s sage, magic mint, lady salvia, or simply salvia, is a plant species with transient psychoactive properties when its leaves containing the opioid-like terpenoids are consumed by chewing, smoking, or as a tea. The native habitat of salvia is the remote and hilly Sierra Mazateca region of Mexico. The authors of [84] introduced the TLC-GC/MS method for rapid screening of herbal products containing *Salvia divinorum* for the contents of salvinorin A, the most potent diterpenoid present in this herb. The TLC analysis was carried out following the procedure incompletely described in [18]. The only known details are that the crushed fresh or dried leaves were extracted with acetonitrile and the TLC analysis was carried out against the salvinorin A standard on the Whatman silica gel pre-coated chromatographic plates.

*Psilocybe mexicana* is a psychedelic mushroom from the family of Hymenogastraceae, genus *Psilocybe*, and its first known usage was by the natives of North and Central America over 2000 years ago, in the so-called mushroom cult. There are also numerous other fungi belonging to the same genus which naturally produce psychoactive compounds psilocybin, psilocin and baeocystin such as, e.g., the tiny *Psilocybe baeocystis* fungus, which is common in the Pacific Northwest. The most potent and the most largely distributed in the temperate regions of the Northern Hemisphere (and particularly in Europe) is *Psilocybe semilanceata*, commonly known as the liberty cap. The first report on the TLC analysis of psilocybin and psilocin contained in *Psilocybe baeocystis* (Singer and Smith) is given in [85]. Frozen fungi were first ground and then macerated in methanol for 12 h, following a well-elaborated working protocol. For the purpose of the TLC analysis, the authors experimented with three different stationary phases, i.e., with silica gel, microcrystalline cellulose and alumina, and with nine different mobile phases. Eventually, the best separation results were achieved on silica gel as stationary phase and with butanol–acetic acid–water (12:3:5, *v*/*v*/*v*) as the mobile phase. Visualization was first performed in the UV light, then the spots were encircled with a pencil, and finally, the plates were sprayed with the visualizing reagent (10% *p*-dimethylaminobenzaldehyde in conc. hydrochloric acid). Thin-layer chromatography was also used to discover a new mushroom from the *Psilocybe* genus, i.e., *Psilocybe germanica* sp. nov. [86]. The authors used their own extraction protocol to isolate compounds of interest from the mushroom, which in principle was also based on methanol as an extractant. For the purpose of the TLC analysis, silica gel was employed as the stationary phase and two mobile phases: (i) *n*-butanol–acetic acid–water (2:1:1, *v*/*v*/*v*) and (ii) methanol–aqueous ammonia (25%) (100:1.5, *v*/*v*) were used, both providing a positive separation result. Detection of the compounds of interest was carried out against the psilocybin, baeocystin and psilocin standards, and it was based on a comparison of the retardation factor (*R*_F_) values for the standards with those for respective fractions derived from the mushroom extract.

Summing up, the contents of this review paper cover a vast area of TLC applications to a large number of important and demanding analytical tasks within the framework of screening botanicals, in which TLC in most cases plays a sovereign and important role. Our review is illustrated by selected cases derived from original research papers and by certain overview papers focused on the narrower thematic scopes. They have been chosen in an arbitrary manner following personal experience and preferences of the authors, as it has been our intent to emphasize recent achievements in the field discussed, yet at the same time to present some of the most interesting and hopefully inspiring cases of the method development. To make it easier for our readers to navigate through the collected material and to facilitate a targeted access to the areas of primary interest, in Table 1, we point out these references cited in our review, which we regard as the most important within each of the seven TLC application types mentioned therein.

## 6. Conclusions

It was an aim of this study to present a multifaceted picture of the huge versatility of thin-layer chromatography in plant research and in solving important and diverse practical tasks, by emphasizing four main directions of its applicability. Two directions, (i) thin layer chromatography in chemotaxonomy of plants and (ii) screening of a wide spectrum of biological properties of plants, have a considerable cognitive potential. The former one is able to contribute to plant chemosystematics and hence to the development of botany, ethnobotany and the related disciplines. The latter one is able to contribute to a wide number of research areas ranging from environmental issues to medicine. Further two applicability directions, (iii) thin layer chromatography in screening of medicinal and culinary herbs, and in quality control of alimentary and cosmetic products of botanical origin and (iv) planar chromatography in screening of psychoactive plants, focus on purely analytical aspects related to the quality control of botanicals, with a need to conform with demands of official regulatory bodies.

Research output in each of the four aforementioned directions differs in terms of the number of published papers and undoubtedly for diverse reasons. An obvious advantage of TLC, which is a relatively low-cost technique that combines high speed and easy availability of conclusive results, enjoys the highest output with papers which introduce new methods of rapid screening medicinal and culinary herbs and spices and quality control of alimentary and cosmetic products. A low output of papers is observed with planar chromatographic screening of psychoactive plants, which is probably due to sensitivity of the issue itself and due to higher rapidity with the other instrumental techniques as an urgent demand for forensic purposes. Chemotaxonomy of plants by means of the TLC methods is represented by a good number of scientific reports, although current trends in this field start reorienting plant taxonomy towards genetic taxonomy, owing to available new analytical tools able to reveal genetic profiles of studied organisms [87,88,89]. Screening of biological (i.e., antioxidant, anti-microbial and enzymes-inhibiting) properties of plants is a quite exciting and relatively new research field based on the TLC methods. It characterizes a steadily growing number of published papers which augurs a very positive further direction of its development.

As indicated by the above-presented reflections, this study has not been planned as a review paper in a traditional sense of the word, but instead, it intentionally presents a diverse mosaic of applications which highlights the most important roles and functions of the TLC in the screening of botanicals, indicates further directions of the development in the field, and provides short selection of adequately chosen and hopefully inspiring illustrative examples. It can also be viewed as a mini-tutorial addressed to younger scientists (and at first instance, to PhD students and postdocs), with an aim to demonstrate the real power of such a modest and therefore sometimes disregarded analytical tool such as thin-layer chromatography, when implemented with the researcher’s knowledge, inventiveness and vision.

## Figures and Tables

**Table 1:** Scoping review of references on thin-layer chromatography (TLC) in screening of botanicals.

Applicability Areas to Screening Botanicals	Selection of Topical Reads
Chemotaxonomy of plants	[6,7,8,9,19,20,21,22,23,24,25,26,27,28,87,88,89]
Screening of botanicals for free radical scavenging activity	[10,11,12,14,31,32,33,34,35,36,37,38,39]
Screening of botanicals for antimicrobial properties	[11,12,14,40,41,42,43,44,45,46]
Screening of botanicals for enzyme inhibiting potential	[12,14], [47,48,49,50,51,52,53,54,55,56,57]
Quality control of medicinal and culinary herbs	[16,58,59,60,61,62,63]
Quality control of alimentary and cosmetic products of botanical origin	[15,17,64,65,66,67,68]
Screening of psychoactive plants	[18,69,70,71,72,73,74,75,76,77,78,79,80,81,82,83,84,85,86]
